# Three Cases of Indirect Decompression Failure Following Oblique Lumbar Interbody Fusion Requiring Early Direct Posterior Decompression: Analysis of Etiologies and Literature Review

**DOI:** 10.7759/cureus.90798

**Published:** 2025-08-23

**Authors:** Satoshi Hattori, Satoru Matsutani

**Affiliations:** 1 Spinal Surgery, Hachioji Spine Clinic, Hachioji, JPN

**Keywords:** cage subsidence, cross-sectional spinal canal area, direct posterior decompression, endplate injury, indirect decompression failure, lumbar spinal canal stenosis, marchi grade, oblique lumbar interbody fusion (olif), schizas grade, surgical complication

## Abstract

We present three cases of indirect decompression failure (IDF) following oblique lumbar interbody fusion (OLIF). Additional direct posterior decompression (DPD) was performed within a week of the initial procedure to alleviate persistent or deteriorating symptoms due to nerve compression. The IDF incidence rate among patients with lumbar spinal stenosis who underwent OLIF at our institution was 1.8% (three out of 211 patients). Two elderly women (aged 80 and 81) sustained an endplate injury (EPI) and a minor vertebral fracture, followed by cage subsidence, due to untreated osteoporosis during the perioperative period. This resulted in a 43% and 10% decrease in the cross-sectional spinal canal area (CSA), respectively, compared to the preoperative value, leading to neurological deterioration. The third case involved a 74-year-old man with bony foraminal stenosis that could not be alleviated by OLIF, resulting in residual radiculopathy. The effectiveness of indirect decompression via OLIF procedures for patients with severe central stenosis remains a topic of debate. If it is supposed to be effective, the absence of subsequent cage subsidence following OLIF is considered a crucial prerequisite. Although bony foraminal and lateral recess stenosis were significant risk factors for IDF following OLIF in our case analysis as in previous reports, there are few effective parameters with which to predict IDF. Further studies should establish quantitative and integrated thresholds for the predictive parameters of IDF, particularly in conjunction with preoperative CSA and cross-sectional foraminal area (CFA), the restoration of posterior disc height (PDH) during surgery, the Marchi grading system for cage subsidence, and the severity of osteoporosis as indicated by the CT-Hounsfield unit values.

## Introduction

A combination of surgical decompression and spinal fusion is used to treat symptomatic lumbar spinal stenosis when dynamic and static stenosis coexist or when specific conditions, such as foraminal stenosis or previous surgery, are present. Lateral lumbar interbody fusion (LIF) using oblique lumbar interbody fusion (OLIF) techniques can provide spinal fusion and decompression simultaneously in an indirect manner [1-3]. However, the conditions necessary for indirect decompression to successfully substitute direct posterior decompression (DPD) remain unclear in terms of both preoperative and intraoperative patient- and surgical procedure-related factors [3-6].

Previously reported incidence rates of indirect decompression failure (IDF) have varied widely across studies (6.4-30%) because the definition of IDF has been inconsistent among them [3-8]. In this report, we defined IDF as 'the presence of persistent or deteriorating nerve compression symptoms following initial OLIF procedures, necessitating additional DPD within three weeks of the initial procedure.' In order to evaluate the efficacy of the inclusion criteria for indirect decompression via OLIF (i.e. the exclusion criteria for DPD), we selected cases of severe IDF that required salvage posterior decompression surgery within three weeks of OLIF. We reported three cases (1.8%) of IDF among the 211 consecutive OLIF cases performed at our institute between June 2019 and July 2024. All three cases met the criteria for this definition. As case reports of IDF describing the underlying pathologies in detail are rare, we analysed the causes of failure and the technical requirements for prevention in these cases. We also reviewed the literature and discussed the limitations of indirect decompression.

## Case presentation

Case 1 

**Figure 1:**
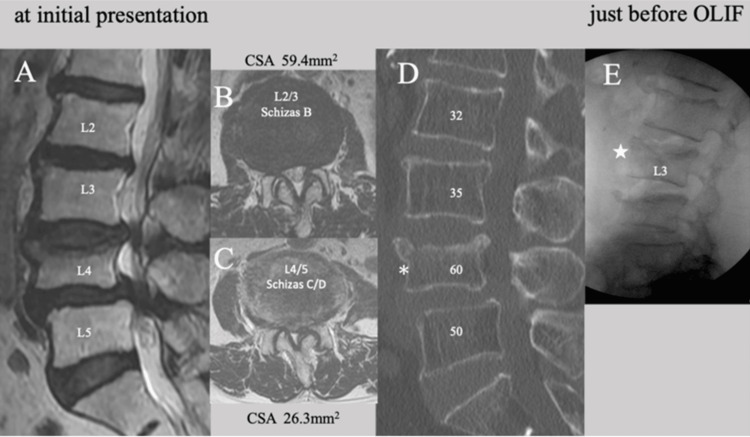
Preoperative MRI and CT images of Case 1 MRI and CT images of an 81-year-old woman (Case 1) showing (A, B) moderate (Schizas B at L2/3) and (A, C) severe canal stenosis (Schizas C/D at L3/4 and L4/5) with an old L4 vertebral fracture (D, asterisk) and degenerative spondylolisthesis, and (D) very low L2-L4 CT-Hounsfield unit values. A lateral lumbar X-ray (E) taken just before OLIF showing a newly onset L3 vertebral fracture (star). OLIF: oblique lumbar interbody fusion, CSA: cross-sectional spinal canal area

An 81-year-old woman complained of low back pain, bilateral lower limb pain with numbness, dysuria, and intermittent claudication within 10 meters for the past year. Magnetic resonance imaging (MRI) revealed moderate lumbar spinal stenosis at L2/3 (Schizas grade B) and severe stenosis at L3/4 and L4/5 (Schizas grade C/D, Figure 1A, 1B, 1C). The computed tomography (CT) image at the initial presentation revealed lumbar spondylosis, L4 anterior and L2 posterior spondylolisthesis, and an old L4 osteoporotic vertebral fracture with mild compression (Figure 1D, asterisk). The CT-Hounsfield unit values of the L2-L4 vertebral bodies were very low, at less than 60, indicating the presence of osteoporosis (possibility of severe osteoporosis, Figure 1D). Although a newly onset L3 vertebral fracture was identified just before the operation, the fracture type was a superior endplate injury (EPI) with minimal depression (Figure 1E, star).

**Figure 2:**
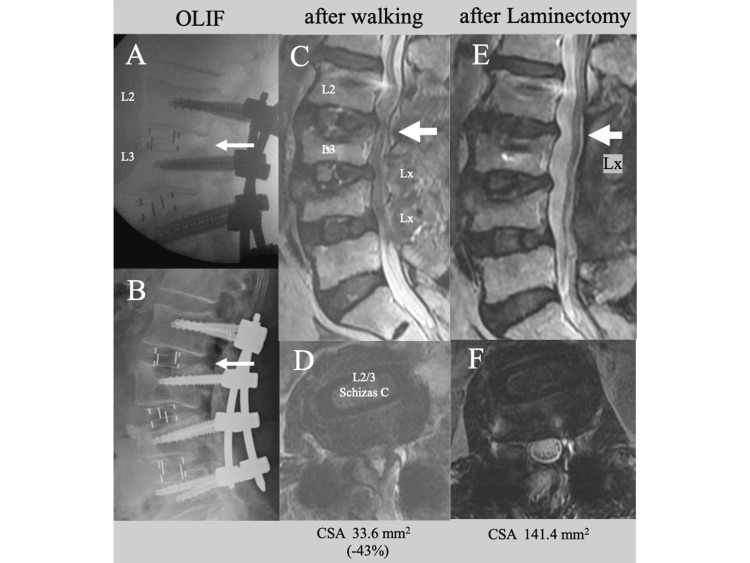
Postoperative lateral lumbar X-rays and MRI images of Case 1 A lateral lumbar X-ray (A) taken immediately after OLIF with pedicle screw fixation showing restoration of PDH at L2/3 (arrow), (B) subsequent advancement in cage subsidence with a decrease in PDH (Marchi grade I, arrow) after commencing walking, and postoperative MRI images showing (C) a narrow dural sac (large arrow) and (D) a 43% decrease in CSA. Following direct posterior decompression at L2/3 (large arrow in E), MRI images (E, F) showing an enlarged dural sac. OLIF: oblique lumbar interbody fusion, PSF: pedicle screw fixation, PDH: posterior disc height, CSA: cross-sectional spinal canal area

Spinal fusion was performed using OLIF at L2-L5, combined with simultaneous laminectomy at L3/4 and L4/5, as well as percutaneous pedicle screw fixation. OLIF cages measuring 10x50x18 mm (6° lordotic CLYDESDALE PTC™ cage, Medtronic, Minneapolis, MN, USA) were inserted at each level. As the central stenosis at the L2/3 level was moderate and the L3 fracture was only a slight central depression of the superior endplate, an indirect decompression effect through restoring posterior disc height (PDH) was anticipated from using a wide, tall OLIF cage (Figure 2A, arrow), without performing additional DPD at this level. However, after starting to walk again following the operation, the patient experienced a deterioration in the numbness and motor weakness (manual muscle test (MMT): quadriceps 4-/4- and tibialis anterior 3-/4) in her lower limbs. A postoperative radiograph revealed advanced cage subsidence at L2/3 with a decrease in PDH (Marchi grade I, Figure 2B, arrow), and an MRI scan showed a decrease in CSA compared to the preoperative value (from 59.4 mm2 to 33.6 mm2, a 43% decrease, Figure 2C, large arrow; Figure 2D). One week later, an additional laminectomy was performed at the L2/3 level to decompress the dural sac (Figure 2E, large arrow; Figure 2F). Following the procedure, the patient's numbness and motor deficit returned to normal.

Case 2

**Figure 3:**
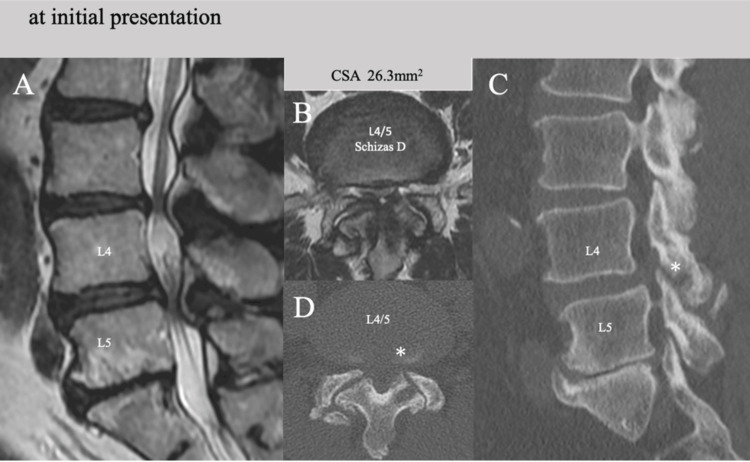
Preoperative MRI and CT images of Case 2 MRI and CT images of a 74-year-old man (Case 2) showing (A, B) moderate to severe canal stenosis at four disc-levels (Schizas D at L4/5) with (C, D) L4 degenerative spondylolisthesis and osteophyte formation of L5 superior facet (asterisks). CSA: cross-sectional spinal canal area

A 74-year-old man presented with severe pain and numbness in his left anterolateral thigh and leg (the areas innervated by the L4-L5 nerve roots), as well as low back pain and intermittent claudication within 100 meters. Radiological imaging of the lumbar spine revealed degenerative spondylosis and spondylolisthesis at L4. An MRI scan confirmed moderate to severe lumbar spinal stenosis at four disc levels: L2/3, L3/4, L4/5, and L5/S1 (Figure 3A). The CSA of the spinal canal at L4/5 was extremely narrow, measuring just 26.3 mm² (Schizas grade D, Figure 3B). Concomitant foraminal stenosis due to osteoarthritic changes, such as superior facet enlargement and osteophyte formation, was also evident at L4/5 (Figure 3C, 3D, asterisks) in CT images.

**Figure 4:**
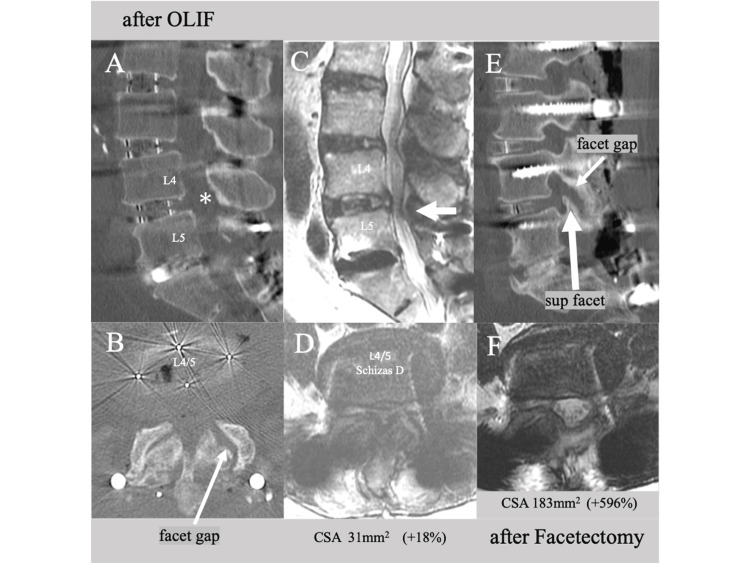
Postoperative CT and MRI images of Case 2 CT images taken immediately after OLIF showing (A) enlarged posterior disc height (asterisk) and (B) increased facet gap (arrow) at L4/5. Postoperative MRI images showing a slight increase in (C) the anteroposterior diameter of the dural sac (large arrow) and (D) CSA (an 18% increase). A parasagittal CT scan after the operation showing (E) residual lateral recess and foraminal stenosis due to a deformed facet at L4/5 (large arrow), despite an increased facet gap (arrow). An axial MRI image taken after a left L4/5 facetectomy (F) showing complete decompression of the L4 and L5 nerve roots and the dural sac. OLIF: oblique lumbar interbody fusion, CSA: cross-sectional spinal canal area

The patient underwent a one-stage operation involving L2-5 OLIF, L5/S posterior lumbar interbody fusion (PLIF), and pedicle and alariliac screw fixation at L2-S2. OLIF cages measuring 10x45x18 mm (6° lordotic CLYDESDALE PTC™ cage) and 10x50x22 mm (12° lordotic CLYDESDALE PTC™ cage) were inserted at L2/3 and L3/4/5, respectively. As no endplate injury or subsequent cage subsidence was observed after OLIF procedures and the disc height was enlarged with increased facet gap at any level (Figure 4A, asterisk; Figure 4B, arrow), direct posterior decompression was not performed. However, he still complained of pain in the left anterolateral thigh and leg after the operation. A postoperative MRI scan revealed insufficient enlargement of the anteroposterior diameter (APD) of the dural sac and CSA (31 mm², an 18% increase, Figure 4C, large arrow; Figure 4D), and residual lateral recess and left foraminal stenosis due to a deformed superior facet at L4/5 (Figure 4E, large arrow). This was despite the enlargement of the posterior disc height and facet gap resulting from the insertion of a 10 mm-high cage (Figure 4B, 4E, arrows). One week later, an additional left L4/5 facetectomy was performed to decompress the L4 and L5 nerve roots. His left anterolateral thigh and leg pain and numbness then disappeared completely.

Case 3

**Figure 5:**
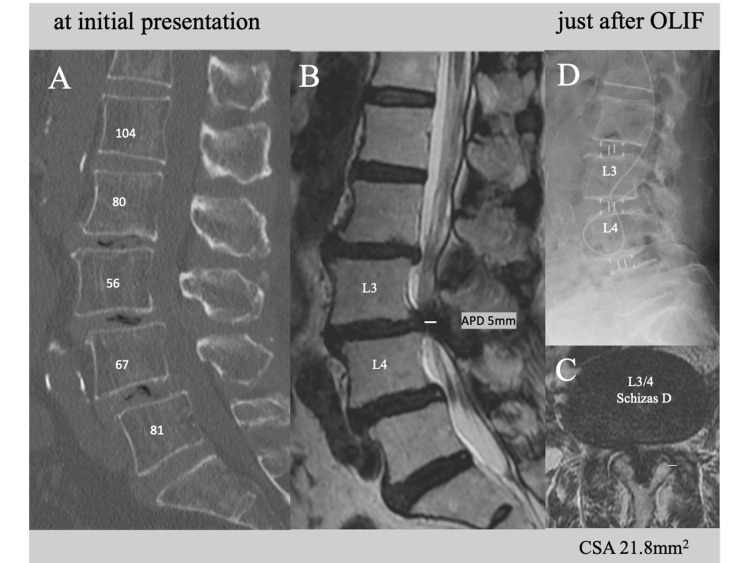
Preoperative CT and MRI images and lateral lumbar X-ray immediately after OLIF of Case 3 MRI and CT images of an 80-year-old woman (Case 3) showing (A, B, C) moderate to severe canal stenosis (Schizas B at L2/3 and L4/5 and Schizas D at L3/4), as well as degenerative spondylolisthesis at L3 and L4, and (B) very low CT-Hounsfield unit values at L2–L4. A lateral lumbar X-ray (D) taken immediately after OLIF showing significant restoration of the posterior disc height and spondylolysis without endplate injury at any level. OLIF: oblique lumbar interbody fusion, APD: anteroposterior diameter, CSA: cross-sectional spinal canal area

An 80-year-old woman complained of severe pain and numbness in her left lateral thigh and leg, as well as muscle weakness (left drop foot) and difficulty in standing and walking (consistent with left L5 radiculopathy). CT images of her lower back revealed degenerative lumbar scoliosis and multiple spondylolisthesis (posterior at L2, anterior at L3 and L4, Figure 5A). An MRI scan revealed moderate lumbar spinal stenosis at L2/3 and L4/5 (Schizas grade B, Figure 5B) and severe stenosis at L3/4 (Schizas grades D, Figure 5B, 5C). The CT-Hounsfield unit values of the L2-L5 vertebral bodies were very low (56-81), indicating the presence of osteoporosis (possibility of severe osteoporosis, Figure 5A). Spinal fusion was performed using OLIF at L2-L5 in the first stage, followed by a laminectomy at L4/5 with percutaneous pedicle screw fixation between L2-L5 three days later. OLIF cages measuring 10x50x18 mm (6° lordotic CLYDESDALE PTC™ cage) were properly inserted, and sufficient posterior disc height restoration was achieved at each level without endplate injury (Figure 5D). There was no deterioration in the preoperative symptoms or neurological status.

**Figure 6:**
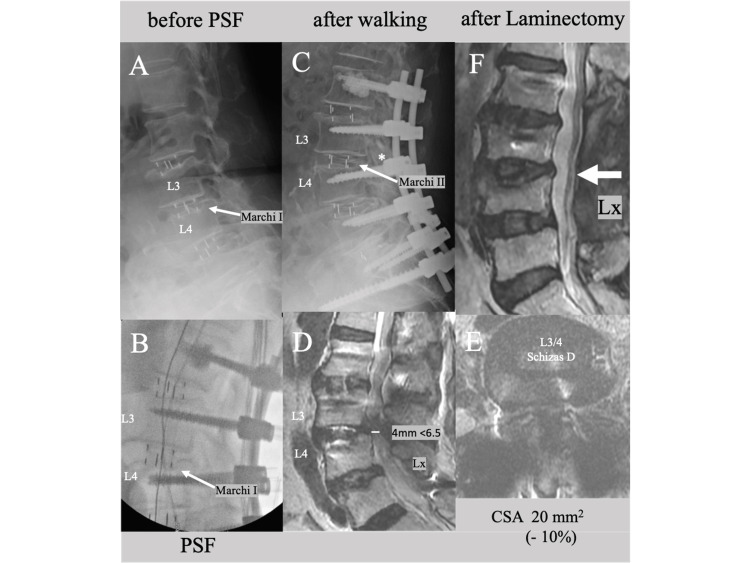
Lateral lumbar X-rays nnd MRI images after OLIF, PSF and an additional laminectomy of Case 3 A lateral lumbar X-ray (A) taken shortly before the second posterior operation (following a fall) showing mild endplate injury and cage subsidence at L3/4 (Marchi grade I, arrow). A lateral lumbar fluoroscopic image during pedicle screw fixation (B) showing no deterioration of cage subsidence. After commencing walking, a lateral lumbar X-ray (C) showing advancement of the cage subsidence (Marchi grade II, arrow), with a decrease in PDH (asterisk) at L3/4. MRI images showing (D) a decrease in the anteroposterior diameter (APD) of the dural sac (from 5 mm to 4 mm) and (E) a 10% decrease in CSA. After performing an additional laminectomy at L3/4, an MRI image (F) showing sufficient enlargement of the dural sac. PSF: posterior screw fixation, PDH: posterior disc height, CSA: cross-sectional spinal canal canal area

Shortly before the second posterior operation, however, she fell due to delirium, resulting in a mild endplate injury and cage subsidence at three disc levels (Marchi grade I at L3/4, Figure 6A, arrow). As the level of the disc responsible for the chief complaint (L5 radiculopathy) was considered to be L4/5 and the EPI was mild with subsequent Marchi grade I cage subsidence, additional direct posterior decompression was not performed at L3/4 during the second-stage operation, despite the severe canal stenosis at L3/4 (Schizas grade D). Cage subsidence did not deteriorate just after pedicle screw fixation (Figure 6B, arrow). However, after commencing walking again following the posterior operation, she experienced a deterioration in numbness and motor weakness in her lower limbs, as well as dysuria, which suggested cauda equina syndrome. The postoperative radiograph revealed advanced cage subsidence at L3/4, progressing from Marchi grade I to grade II (Figure 6C, arrow), as well as no increase in PDH compared to the preoperative value (Figure 6C, asterisk). The MRI scan showed a decrease in the APD of the dural sac from 5 mm to 4 mm (Figure 6D) and a decrease in the CSA from 21.8 mm² to 20 mm² (a 10% decrease; Figure 6E). One week later, an additional laminectomy at L3/4 level was performed to decompress the spinal canal and foramina (Figure 6F). Following the procedure, her numbness and motor deficit improved, and she was able to walk with a T-cane.

Table 1 provides the detailed demographic data for the three illustrative cases.

**Table 1:** Demographic data of illustrative three cases DL: degenerative listhesis, VFx: vertebral fracture, DLS; degenerative lumbar scoliosis, OP: osteoporosis, LBP: low back pain, CES: cauda equina syndrome, IMC: Intermittent claudication, IDF; indirect decompression failure, OLIF: oblique lumbar interbody fusion, CSA: cross-sectional spinal canal area, AC/AP: anterior edge-center/anterior-posterior edge of the OLIF cage at sagittal plane, PDH: posterior disc height, DEXA: dual energy X-ray absorptiometry, %YAM: % young adult mean, CT-HU value: computed tomography-Hounsfield unit value, EPI: endplate injury, PDD: posterior direct decompression, ID: indirect decompression, Lx: laminectomy Two data sets of CSA, Schizas grade, and PDH showed preoperative (left) and postoperative (right) measurements, respectively.

	Case 1	Case 2	Case 3
Age	81	74	80
Sex	woman	Man	woman
Disease	DL (retrolisthesis)	DL	DL
	VFx (L3)	Facet degeneration	DLS
	OP		OP
Chief complaint	LBP	LBP	LBP
	CES	Lt L4L5 Radiculopathy	Lt L5 Radiculopathy
	IMC (10m)	IMC (100m)	Difficulty of standing & walking
			drop foot
Level of OLIF	L2/3/4/5	L2/3/4/5	L2/3/4/5
Level of DPD	L3/4/5		L4/5
Level of indirect decompression (ID)	L2/3	L2/3/4/5	L2/3/4
Level of IDF	L2/3	L4/5	L3/4
CSA (mm^2^)	59.4→33.6	26.3→31	21.8→20
Schizas grade	B→C	D→D	D→D
% change of CSA	-43%	+18%	-10%
Cage size	10x50x18mm	10x50x22mm	10x50x18mm
Cage angle	6°	12°	6°
Cage sagittal center	39% (AC/AP)	43%	46%
Cage subsidence	(+)	(-)	(+)
Marchi grade	II	0	II
PDH (mm)	3.5→3.2	7.2→8.0	5.0→5.0
DEXA T-score	-3.9T (L2), -3.4T(L3)	3.9T (L4)	1.0T (L3), 3.3T (L4)
CT-HU value	32 (L2), 35 (L3)	128 (L4), 150 (L5)	56 (L3), 67 (L4)
BMI	21.9	27.9	28.2
Posterior operation	Laminectomy (L2/3)	Facetectomy (L4/5 Lt)	Laminectomy (L3/4)
following OLIF	1W	1W	1W
Outcome	improvement of CES	improvement of radiculopathy	improvement of CES+radiculopathy
Cause of IDF	VFx/EPI/OP	foraminal stenosis	EPI after fall/OP
	cage subsidence	osteophyte formation	cage subsidence

## Discussion

OLIF has been widely used to treat degenerative lumbar disorders since the early 2010s with the development of the OLIF25™ and OLIF51™ systems [1]. Among several advantages of OLIF, indirect decompression is the most characteristic effect of OLIF, as well as minimal invasiveness and strong deformity correction with a high fusion rate [1,2]. Restoring disc height and correcting deformities such as scoliosis, slippage and axial rotation of the spine using the large footprint cage can increase the central canal and foraminal areas by up to 30-50% immediately after surgery [9]. The CSA increases by up to 100-150% after two years [9]. Previous studies have shown that the outcomes of indirect decompression are not inferior to those of direct posterior decompression in terms of pain scale and radiological parameters [5]. Moreover, OLIF is becoming increasingly popular due to its ability to decrease blood loss and pain, avoid dural tears and nerve damage, and reduce the risk of complications in patients who have undergone previous surgeries [10,11], as well as in elderly patients with severe deformities [1-3].

However, it remains unclear which patients are suitable candidates for indirect decompression and which require DPD following LIF. In the present background case series, indirect decompression was performed on 85% of patients and 90% of the operated disc levels. The inclusion criteria for DPD in the present OLIF case series were defined as follows: 1) severe motor deficit (MMT <4); 2) neurogenic bladder or bowel dysfunction; 3) static clinical symptoms with persistent pain and numbness in the lower extremities at rest more than 50% of those while standing or walking; and 4) radiological evidence of severe static stenosis due to a large extruded disc herniation, a large facet cyst, or bony stenosis. We did not impose any limitations on performing indirect decompression in patients with severe spinal stenosis of Schizas grades C and D unless these inclusion criteria for DPD were absent. Over-indication of indirect decompression could lead to IDF. Conversely, under-indication of indirect decompression could result in extra DPD, thereby reducing the benefits of LIF.

The incidence of IDF following LIF, with or without the need for additional posterior decompression surgery, varies widely across previous reports (6.4-30%) due to differences in IDF definition [5-8]. Furthermore, some authors have emphasised the under-reporting of IDF [7]. In this case series, IDF was observed in only 1.8% (3/211) of indirect decompression patients following OLIF, which is considered relatively rare compared to the previous studies [4]. The low incidence of IDF in our case series may be primarily due to our inclusion criteria, which required additional posterior decompression surgery in the early period after OLIF. However, by the final follow-up after one year, none of the 211 patients, except the three present cases, required additional posterior decompression surgery, so the current inclusion criteria for indirect decompression were considered rational.

Previous reports have discussed both patient- and surgical procedure-related factors that induce IDF in LIF. Patient factors include static clinical symptoms [12], muscle weakness of less than 4 on MMT scale [12], locked disc height (a locked facet joint) [3,12,13], static stenosis (e.g., bony foraminal stenosis, a free disc fragment or a facet cyst) [7,8,12-14], congenital or severe spinal stenosis [6,12,13], and osteoporosis [3,12,15]. Procedure-related factors include anterior cage placement in the vertebral column [16], severe cage subsidence (Marchi grades II-III) [3], and insufficient restoration of posterior disc height (<10 mm) [3]. The present inclusion criteria for DPD are consistent with these predictive factors [4] and are considered reasonable (Table 2). 

**Table 2:** Candidates for the predictors of IDF involved in the present criteria and previous reports IDF: indirect decompression failure, MMT: manual muscle test, BBD: bladder and bowel dysfunction, CSA: cross-sectional spinal canal area, APD: anteroposterior diameter, EPI: endplate injury, LIF: lateral interbody fusion, A-P: anteroposterior

Patient factors		References
static clinical symptoms	>50% pain at rest compared to the time while walking	[12]
motor weakness	MMT <4/5	[12]
locked disc height	<1mm change from upright to supine (locked facet)	[3,12,13]
static stenosis	bony foraminal & lateral recess stenosis	[7,8,12-14]
	free disc fragment	[12,13]
	facet cyst	[12,13]
lateral recess depth	<3mm	[20]
severe stenosis	Schizas’ grades C and D	[6,12]
	CSA <34.4 mm^2 ^(MRI), <44 mm^2^ (MRI), <80 mm^2^ (MRI)	[18-20]
	AP Dural Diameter <6.5 mm (MRI)	[18]
	CSA <80 mm^2^ (CT)	[20]
congenital stenosis		[12,13]
osteoporosis	T-score < -2.1T ~ -2.5T (DEXA)	[3,12,15]
Surgical & Technical factors		
cage placement	anterior of the vertebral body	[16]
posterior disc height	<10 mm	[3]
EPI & cage subsidence	Marchi’s Grade II-III	[3]
	tall LIF cage >12 mm height	[6,12,15]
	narrow LIF cage <22 mm width (A-P)	[6,12,15]
	concave endplate morphology in the sagittal plane	[17]

With regard to EPI after OLIF procedures, a cage size of >12 mm in height and <22 mm in width [6,12,15], concave endplate morphology in the sagittal plane [17], and a low T-score [3,12,15] have been reported as risk factors for EPI and subsequent cage subsidence (Table 2). In our previous study, the incidence of EPI with cage subsidence of over 2 mm was found to be 9% during OLIF procedures, rising to 20% within two months of surgery, and significant risk factors for EPI were found to be tall and narrow-width cage size, high body mass index (BMI) and low T-score (Hattori S, et al., 2020, presented at the 29th Annual Meeting of the Japanese Spinal Instrumentation Society). These results suggest that early cage subsidence is not uncommon, even with the insertion of a large-footprint cage in OLIF.

Currently, there is insufficient consensus regarding the correlation between the preoperative radiological severity of stenosis and clinical outcomes following indirect decompression with OLIF. Lu et al. reported that indirect decompression using the OLIF technique could not achieve significant clinical improvement in cases of severe central spinal canal stenosis with CSA below 33.4 mm² and could only achieve limited improvement in cases with CSA below 75 mm², as measured by MRI [18]. Also, Wu et al. and Bokov et al. reported that severe stenosis with CSA below 44 and 80 mm² as measured by MRI and CT, respectively, was associated with IDF [19,20]. Meanwhile, Schimizu et al. reported positive clinical outcomes following OLIF indirect decompression even in cases of severe stenosis graded C and D according to the Schizas classification system [9].

As the radiological effects of indirect decompression in OLIF are generally considered to be smaller than those of posterior direct decompression, the clinical effects of OLIF may primarily stem from its strong fusion properties rather than from indirect decompression itself. Pseudoarthrosis of the OLIF cage can lead to cage subsidence, reducing both PDH and CSA below the levels achieved immediately following OLIF insertion. Furthermore, in patients with moderate to severe stenosis (Schizas grades B-C/D), severe cage subsidence exceeding Marchi grade II can decrease PDH and CSA to below preoperative levels, leading to a deterioration in neurological function, as was observed in cases 1 and 3. In this context, achieving complete fusion of the OLIF cage is crucial for better clinical outcomes, particularly in terms of preventing serious cage subsidence and a decrease in PDH and CSA to below preoperative levels in cases of severe stenosis, as well as providing segmental stabilisation. 

Cases 1 and 3 illustrate the difficulties in predicting the outcomes of indirect decompression when dealing with EPI, cage subsidence, osteoporosis and moderate to severe central canal stenosis prior to surgery. Despite wide-width PTC cages being inserted into the middle portion of the affected levels, these cages sank further than expected once the patients started walking. This resulted in a rapid deterioration in pain and neurological function due to a decrease in CSA. Thorough evaluation of osteoporosis is also important in order to predict the substantial risk of EPI and subsequent progression of cage subsidence.

Case 2 illustrates the challenge of predicting the indirect decompression effect in patients with bony stenosis, even when a cage exceeding 10 mm in height is inserted, due to the limited number of parameters available for quantitatively assessing the levels of bony lateral or foramen stenosis. Several authors have reported that foraminal stenosis accompanied by osteophyte formation or superior facet hypertrophy increases the risk of IDF following LIF, and have recommended simultaneous posterior decompression [7,8,12,14,20]. However, there are few quantitative preoperative parameters with which to evaluate the threshold of foraminal bony stenosis at which DPD should be performed.

The CSA, cross-sectional foraminal area (CFA) and APD of the dural sac on the preoperative MRI scan, the restoration of the PDH on the intraoperative X-rays, and the grade of Marchi's cage subsidence could be practical, quantitative parameters with which to predict IDF, even in complex cases such as the present three IDF cases. It is also important to fully evaluate osteoporosis using Hounsfield unit values on CT scans, in addition to dual energy X-ray absorptiometry (DEXA) T-scores, to predict the perioperative risk of EPI and subsequent cage subsidence. Further prospective, quantitative studies are needed to avoid IDF and reduce the need for further unexpected surgery.

## Conclusions

IDF occurred in three out of 211 patients (1.8%) undergoing OLIF surgery for degenerative lumbar disease. The causes of IDF were: 1) an EPI and a minor vertebral fracture with subsequent cage subsidence in two patients with untreated osteoporosis and moderate to severe central spinal canal stenosis, which resulted in a 43% and 10% decrease in CSA compared to the preoperative value, leading to neurological deterioration; and 2) underdiagnosed bony lateral recess and foraminal stenosis in one patient, which could not be alleviated by indirect decompression via OLIF, resulting in residual radiculopathy. The effectiveness of indirect decompression via OLIF procedures for patients with severe central stenosis remains a topic of debate. If it is supposed to be effective, the absence of subsequent cage subsidence following OLIF is considered a crucial prerequisite. Although bony foraminal and lateral recess stenosis were significant risk factors for IDF following OLIF in our case analysis as in previous reports, there are few effective parameters with which to predict IDF. Further studies should establish quantitative and integrated thresholds for the predictive parameters of IDF, particularly in conjunction with preoperative CSA and CFA, the restoration of PDH during surgery, the Marchi grading system for cage subsidence, and the severity of osteoporosis as indicated by the CT-Hounsfield unit values.
